# Tattoo Ink Metal Nanoparticles: Assessment of Toxicity In Vitro and with a Novel Human Ex Vivo Model

**DOI:** 10.3390/nano15040270

**Published:** 2025-02-11

**Authors:** Beatrice Battistini, Daniela Lulli, Beatrice Bocca, Maria Luigia Carbone, Carmela Ramondino, Stefano Caimi, Alessio Capone, Ezio Maria Nicodemi, Elena Dellambra, Isabella De Angelis, Cristina Maria Failla

**Affiliations:** 1 Department of Environment and Health, Istituto Superiore di Sanità, 00161 Rome, Italy; beatrice.battistini@iss.it (B.B.); beatrice.bocca@iss.it (B.B.); stefano.caimi@iss.it (S.C.);; 2 Experimental Immunology Laboratory, IDI-IRCCS, 00167 Rome, Italy; d.lulli@idi.it (D.L.); marialuigia.carbone@idi.it (M.L.C.); c.ramondino@idi.it (C.R.); c.failla@idi.it (C.M.F.); 3 Molecular and Cell Biology Laboratory, IDI-IRCCS, 00167 Rome, Italy; a.capone@idi.it; 4 Department of Plastic Surgery, IDI-IRCCS, 00167 Rome, Italy; e.nicodemi@idi.it

**Keywords:** tattoo, metal, nanoparticle, toxicity

## Abstract

Tattoo inks contain varying amounts of metal nanoparticles (NPs) < 100 nm that, due to their unique physicochemical properties, may have specific biological uptake and cause skin or systemic toxicities. The toxic effects of certified reference standards of metal NPs and samples of commercially available tattoo inks were investigated using an in vitro system and a novel human ex vivo model. In vitro toxicity was evaluated using vitality assays on human skin cells (HaCaT cell line, primary fibroblasts, and keratinocytes). No toxicity was observed for Al_2_O_3_, Cr_2_O_3_, Fe_2_O_3_, and TiO_2_ NPs, whereas CuO NPs showed dose-dependent toxicity on HaCaT and primary fibroblasts. Fibroblasts and keratinocytes were also sensitive to high concentrations of ZnO NPs. Reference standards and ink samples were then injected ex vivo into human skin explants using tattoo needles. Histological analysis showed pigment distribution deep in the dermis and close to dermal vessels, suggesting possible systemic diffusion. The presence of an inflammatory infiltrate was also observed. Immunohistochemical analysis showed increased apoptosis and expression of the inflammatory cytokine interleukin-8 in explants specifically tattooed with the reference standard or red ink. Taken together, the results suggest that the tattooing technique leads to exposure to toxic metal NPs and skin damage.

## 1. Introduction

Historically, the practice of tattooing remained limited to certain individuals, such as sailors, soldiers, prisoners, or criminals. Then, in the 1970s and 1980s, tattooing began to be practiced by other groups as a symbol of rebellion against society. In recent years, tattooing has had an exponential increase, especially among the youth [1,2]. Regulatory laws have been proposed to improve the safety of tattooing [2] based on market surveillance data that provide knowledge on hazardous substances present in tattoo inks and on clinical data that correlate tattooing with adverse skin reactions [3,4,5]. Common dermatological complications of skin tattooing are represented by allergic reactions, mostly elicited by specific pigments and resistance to corticoid treatment [6]. Papulo-nodular granulomatous reactions are also frequently detected, whereas autoimmune reactions such as psoriasis vulgaris, lichen planus, cutaneous lupus erythematosus, and vitiligo have been more rarely reported [2]. Development of skin cancer in the tattooed area has been reported, but it is considered a rare and rather coincidental event [7,8]. The possible carcinogenic effect of tattoo inks remains unproven.

Tattoo inks are mainly composed of organic pigments, but metals are still among the major ink components, either as chromophores or additives for shading effects or as impurities. Significant concentrations of metal nanoparticles (NPs) such as Cr, Cu, Pb, and Zn NPs have been found in tattoo inks on the market in Italy [9,10]. The metal NP concentrations of known allergens such as Cr, Ni, and Co are often well above the safety limits [11]. Particular concerns arise due to the presence of high amounts of hexavalent Cr, which is carcinogenic and a dermal sensitizer [12]. It should also be considered that the exposure to the tattoo ink is permanent and continuous, with possible development of adverse effects in the long term. Moreover, the smallest NPs can easily penetrate deep into the skin, and tattoo pigments have been found in the lymph nodes of tattooed individuals [13,14]. The action of the immune cells contributes to decrease the tattoo ink from the skin and its transportation to the lymph nodes or distal organs [15]. However, a recent study showed in a murine model that an iron oxide tattoo pigment was minimally distributed from the skin to the liver, and it did not reach the kidney or the brain [16].

Despite the possible systemic absorption of tattoo pigments, none of the chemicals in tattoo ink are formulated for intradermal use. Most inks are composed of pigments that are used in food and beverages and have been approved by regulations such as the European Registration, Evaluation, Authorization, and Restriction of Chemicals (REACH, EC No. 1907/2006) after excluding only oral toxicity. It should also be considered that current regulations mainly address the microbiological and chemical risks associated with tattoo inks, and nanotoxicology is largely overlooked [17].

The relative presence of metal NPs in tattoo inks is highly variable between colors and brands [18], and assessment of tattoo ink risks remains a challenge due to the lack of in vivo data aimed at deeply analyzing the cutaneous and systemic effects of tattoo inks. Different in vitro models have been developed and evaluated, but a consensus was not obtained. Therefore, we tested the toxicity effects of either standard metal NPs or tattoo inks in vitro, in skin cell cultures, and ex vivo in a newly developed method based on tattooed human skin explants.

## 2. Materials and Methods

Physicochemical characterization of NPs. The metal NP reference standards used for the study were as follows: 20 nm Ag (HiQ-Nano, Arnesano, Italy); 5 nm Au, and <100 nm TiO_2_ (Sigma-Aldrich Inc., St. Louis, MO, USA); 30 nm Al_2_O_3_, 60 nm Cr_2_O_3_, 25–55 nm CuO, 30 nm Fe_2_O_3_, and 30–40 nm ZnO (US Research Nanomaterials Inc., Houston, TX, USA). Three different colored inks available in Italy were selected: Mario’s Blue, Light Green, and Bright Red (Solong Tattoo, Shenzhen, China). The dispersions of metal NPs and the selected tattoo inks were characterized by dynamic light scattering (DLS) and single particle mass spectrometry (SP ICP-MS). DLS analysis (Zetasizer Nano-ZS, Malvern Instruments Ltd., Malvern, UK) measured the average hydrodynamic particle size (Z-average in nm) and the size distribution (polydispersity index, PDI) using the cumulated analysis (ISO 22412:2017, https://www.iso.org/obp/ui#iso:std:iso:22412:ed-2:v1:en, accessed on 16 December 2019). Metal NP reference standards were spiked at a concentration of 10 µg/mL in ultrapure deionized water and in Dulbecco’s Modified Eagle Medium (DMEM, Lonza^®^, Milan, Italy) supplemented with 10% fetal bovine serum (FBS). The spiked samples were sonicated for 10 min in an ice-cooled ultrasonic bath, vortexed for 1 min, and then analyzed immediately (T0) and after 24 h (T24). One milliliter of the sample was placed in disposable polystyrene cuvettes and analyzed at 25 °C in the sample chamber after 300 s of equilibration. For DLS measurements, the refractive index and viscosity of the dispersants (water and DMEM) were used, while the specific refractive index and absorption of the metal were applied for spiked samples. The same methodology was applied to tattoo inks after dilution (1:1000) and filtration (0.22 μm) assuming the refractive index and absorption of polystyrene. The reliability and quality of the DLS measurements were controlled by using an automatic attenuator (kept between 7 and 9) with the intercept autocorrelation function set at <0.9 [19], according to the recommendations given in the EUNCL-PCC-001 method [19].

The iCAP Q Inductively Coupled Plasma Mass Spectrometry (ICP-MS) (Thermo Fisher, Bremen, Germany) in single particle (SP) mode and Thermo Scientific™ Qtegra software were used to determine the diameter of the 10 µg/mL-spiked water and the DMEM samples contextually analyzed by DLS. Before the SP ICP-MS analysis, the spiked samples were further diluted with high-purity deionized water to the optimal analytical concentration. Samples were sonicated for 10 min in an ultrasound ice-cooled water bath and vortexed for 1 min to prevent particle agglomeration. To calculate the sensitivity of the ICP-MS system, single-element stock solutions at an analytical concentration of 1 µg/L were used (CPAChem, C.P.A. Ltd., Stara Zagora, Bulgaria). To evaluate the transport efficiency of the ICP-MS sample introduction system, replicate measurements were performed on a Au NP reference standard at a nominal size of 60 nm and a concentration of 19,000 particles/mL (Sigma-Aldrich, St. Louis, MO, USA). The isotopes ^107^Ag, ^197^Au, ^27^Al, ^52^Cr, ^63^Cu, ^56^Fe, ^48^Ti, and ^66^Zn were used for quantification; a dwell time of 5 ms and an analysis time of 60 s per sample were applied [20]. Details of the instrument settings for the DLS and SP ICP-MS analyses of the certified reference standards and tattoo inks are reported in Table 1.

In vitro toxicity assay. A colorimetric method for determining cell viability was performed (MTS assay) for each metal NP reference standard using the CellTiter 96^®^ AQueous One Solution Cell Proliferation Assay (Promega Corporation, Madison, WI, USA). The MTS assay is considered the best choice for metal and metal oxide NMs. It was performed according to the SOPs developed in the NANoREG project and in the EU NanoValid project (https://cordis.europa.eu/project/id/263147/reporting/it, accessed on 16 December 2019). This assay was validated in a European inter-laboratory comparison study [21] and published in an ISO document (ISO—International Standard 19007:2018(E), https://www.iso.org/standard/63698.html#:~:text=ISO%2019007%3A2018%20specifies%20a,variability%20in%20the%20assay%20results, accessed on 16 December 2019).

The human keratinocyte cell line HaCaT (ATCC^®^, PCS-200-011) and primary cultures of human keratinocytes and fibroblasts, previously isolated in our institute from donated biopsies from healthy individuals (Ethical Committee protocol n. 581/3, 2019), were used. HaCaT cells and primary human fibroblasts were maintained in culture with DMEM supplemented with 10% FBS, 1% glutamine (Sigma-Aldrich), and 1% antibiotics (10,000 units/mL streptomycin and 10,000 units/mL penicillin) (Sigma-Aldrich) at 37 °C with 5% CO_2_. Primary human keratinocytes were cultured on a feeder layer of sub-lethally irradiated 3T3-J2 murine fibroblasts as described [22]. Keratinocytes were grown in a 3:1 mixture of DMEM and Ham’s F12 medium (Lonza, Basel, Switzerland), supplemented with 5% HyClone serum (HyClone Laboratories, Inc., Logan, UT, USA), 1 μM hydrocortisone (Calbiochem, Darmstadt, Germany), 1 μM isoproterenol (Sigma-Aldrich), and 0.1 μM insulin (Sigma-Aldrich). For primary cells, experiments were carried out on secondary and tertiary cultures. Cells were grown up to 70–80% confluence. Cells were detached and seeded in 96-well plates at an initial density of 1.8 × 10^4^ cells/well for HaCaT and 2 × 10^4^ cells/well for primary human fibroblasts and keratinocytes. After 24 h, the medium was removed and replaced with a medium containing metal NPs or a dilution of the colored ink. For each experiment, NP concentrations of 0.1, 1, 10, 25, and 50 μg/mL were used diluted in the complete medium. In the positive control wells, the medium was removed and replaced with CdSO_4_ at a concentration range of 9.4–150 μM as a toxicity-positive control. The CdSO_4_ concentration range was chosen based on previous MTS assays performed on the three cell types. After 24 h, the incubation medium was removed and 150 μL of MTS solution in 9.5 mL DMEM was added to each well. Assays were allowed to develop for 1 h. Absorbance at 490 nm was measured with an iMark microplate absorbance reader (BioRad Laboratories, Inc., Hercules, CA, USA). The three selected colors of tattoo inks were also tested following the same procedure described above.

Ex vivo skin tattooing. Human skin explants were donated by healthy individuals who underwent skin tissue removal for plastic surgery. Each person signed an informed consent form, and the study was previously approved by the IDI-IRCCS Ethical Committee (protocol no. 581/3, 2019). The adipose subcutaneous tissue was removed, and the explants were maintained in 1x PBS with the addition of an antibiotic and antimycotic solution (Antibiotic-Antimycotic Solution Stabilized 100X, Sigma-Aldrich). A tattoo rotary machine equipped with disposable tattoo needles (1207M1 and 1205RS) was used (Tattoo machine, Solong Tattoo). Selected colored inks were injected into the skin explants (Mario’s Blue, Light Green, and Bright Red; Solong Tattoo). Negative controls were obtained by analyzing untreated explants or by injecting 1x PBS to evaluate the mechanical damage. CuO NPs (25–55 nm) were also applied at two different concentrations (10 μg/mL and 10 μg/L) as a reference standard. The tattooed samples were then incubated in keratinocyte growth medium (KGM, Lonza) at 37 °C with 5% CO_2_. After 24 h, the medium was refreshed. After an additional 48 h, sections were obtained from each sample and analyzed. The ex vivo tattooed sections were analyzed by SP ICP-MS as summarized in Table 1 [20]. Alkaline extraction was performed using 1.5 mL tetramethylammonium hydroxide (TMAH, Sigma-Aldrich) solution at 25% *v*/*v*. Samples were sonicated for 1 h in an ultrasonic ice-cooled water bath and left at room temperature for 24 h. Subsequently, solutions were filled up to 10 mL with a water solution of 0.1% *v*/*v* Triton X-100 (Alfa Aesar, Ward Hill, MA, USA). Before the analysis, the extraction solutions were further diluted 1:500 with ultrapure deionized water. The medium samples were diluted up to 10 mL with ultrapure deionized water, vortexed for 1 min, and further diluted 1:500 before SP ICP-MS analysis.

Histology and immunohistochemistry. For the histological analysis, staining with a hematoxylin and eosin solution was performed. Sections were dewaxed, rehydrated, and stained for four minutes with Mayer’s Hematoxylin (cod. HMM500, ScyTek Laboratories, Logan, UT, USA). After several washes in distilled water, they were stained for ten seconds with Bluing Reagent (cod. BRT500, ScyTek Laboratories) and then stained for 3 min with Eosin Y Solution (cod. EYB500, ScyTek Laboratories). Finally, the sections were dehydrated in three changes of absolute alcohol and xylene and mounted in mounting medium (EUKITT 09-00250. Bio-Optica, Milan, Italy). For the detection of apoptotic cells, a TdT-mediated dUTP-biotin nick end labeling (TUNEL) assay was used. Sections were dewaxed, rehydrated, fixed with formaldehyde, and then permeabilized with ethanol to allow penetration of the TUNEL reaction reagents into the cell nucleus. Following fixation and washing, the incorporation of biotinylated-dUTP onto the 3′ ends of fragmented DNA was carried out in the reaction containing the TdT enzyme. Incorporated biotinylated-dUTP was visualized by light microscopy following staining with a horseradish peroxidase-conjugated avidin–biotin complex in conjunction with a 3,3′-Diaminobenzidine (DAB) staining solution (Vector Laboratories). Stained sections were analyzed with an AxioCam digital camera coupled with an Axioplan 2 microscope (Carl Zeiss AG). For immunohistochemistry analysis, four-micrometer paraffin-embedded sections of skin explants were obtained, dewaxed, and rehydrated. After quenching endogenous peroxidase, performing antigen retrieval, and blocking non-specific binding sites, the sections were incubated with the following anti-human antibodies: anti-interleukin (IL)-1β antibody (cod. Ab-156791, Abcam; dilution 1:100) and goat polyclonal anti-IL-8 antibody (cod. AF-208-N, R&D Systems, Minneapolis, MN, USA, MSP; dilution 1:20). The sections were incubated overnight at 4° C in a humid chamber. Secondary biotinylated polyclonal antibodies and staining kits were obtained from Vector Laboratories (Burlingame, CA, USA). Immunoreactivity was visualized using a peroxidase reaction with 3-amino-9-ethylcarbazole (AEC) in H_2_O, and the specimens were counterstained with hematoxylin. As a negative control, the primary antibody was omitted. The stained sections were analyzed with the AxioCam digital camera coupled to the Axioplan 2 microscope (Carl Zeiss AG, Oberkochen, Germany).

Statistical analysis. Statistical analysis was conducted using the two-tailed paired Student *t*-test. Statistical significance was set at *p* < 0.05. All statistical analyses were conducted using GraphPad Prism software 7 (La Jolla, CA, USA).

## 3. Results

### 3.1. Measurements of Metal NP Certified Reference Standards

The certified reference standards of Ag, Al_2_O_3_, Au, Cr_2_O_3_, CuO, Fe_2_O_3_, TiO_2,_ and ZnO NPs were measured by DLS and SP ICP-MS to detect their size, size distribution, and stability (after 24 h) (Table 2). The Z-average values were generally higher than the certified diameters provided by the manufacturer. The only exception was represented by Ag NPs, certified at 20 nm, which showed a size of 24.9 nm in water and 20.2 nm in DMEM. The Z-average values of all NPs were comparable in DMEM and water, showing no effects of proteins or other components of the medium on the size of the particles. The exception was ZnO particles, which showed (30–40 nm) a size of 432 nm in water and 43.7 nm in DMEM. The NPs’ size stability in DMEM over 24 h was also investigated, which is the effective exposure time used for in vitro cell experiments. The results showed that NPs’ size tended to decrease at 24 h (e.g., Cr_2_O_3_ and TiO_2_ NP sizes decreased from >100 nm to 86.4 nm and 80.6 nm, respectively). The PDI values obtained ranged between 0.338 (CuO) and 0.743 (TiO_2_) in DMEM at the starting time (T0) and between 0.238 (Ag) and 0.727 (Cr_2_O_3_) in DMEM at T24. In general, the PDI values were over 0.5, indicating poorly dispersed solutions in both water and DMEM. Previous research reported that PDI values < 0.7 were still sufficient to obtain reliable results [23]. The SP ICP-MS results were more consistent with the certified reference diameters, demonstrating this method’s higher accuracy, with an exception for Au found at 27.3 nm (certified, 5 nm) (Table 2).

### 3.2. Presence of Metal NPs in Tattoo Inks

Metal NPs in the Bright Red, Light Green, and Mario’s Blue tattoo inks were analyzed by both DLS and SP ICP-MS (Table 3). The Z-average values were 206.7 nm, 193.4 nm, and 192.8 nm in the red, green, and blue inks, respectively, and the PDI values varied from 0.238 to 0.347. SP ICP-MS identified both the composition of NPs and the size of NPs in the tattoo inks. Table 3 shows that Cr_2_O_3_ and Fe_2_O_3_ NPs with diameters of less than 100 nm were present; CuO NPs were found only in the blue and green inks, with diameters of ca. 50 nm, whereas ZnO NPs were detected only in the red ink with a diameter of 26 nm. Particles of Al_2_O_3_ and TiO_2_ showed diameters of ca. 120 nm and 200 nm, respectively, while Ag and Au NPs were not detected in the three inks examined.

### 3.3. Toxicity of CuO and ZnO NPs in Skin Cells

**Figure 1:**
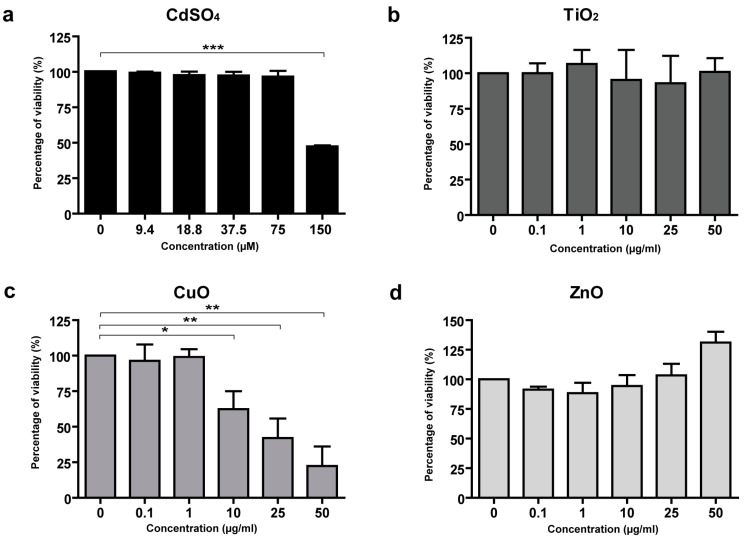
MTS assay evaluating the toxicity of NP standards in the HaCaT cell line. CdSO_4_ was the positive control (**a**). In this cell type, no toxicity was observed with TiO_2_, and ZnO NPs (**b**,**d**), whereas a dose-dependent toxicity of CuO NPs was observed (**c**). Data are expressed as mean values obtained in different experiments ± standard error (SE). * *p* ≥ 0.1. ** *p* ≥ 0.05. *** *p* ≥ 0.01.

**Figure 2:**
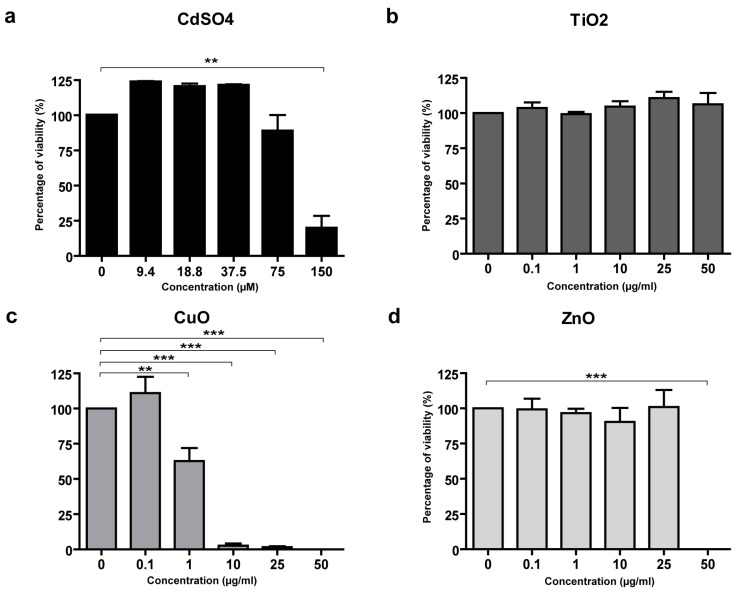
MTS assay evaluating the toxicity of NP standards in primary human fibroblasts. CdSO_4_ was the positive control (**a**). No toxicity was observed with TiO_2_ NPs (**b**), whereas a dose-dependent toxicity was seen with CuO NPs, and with the highest concentration of ZnO NPs (**c** and **d**, respectively). Data are expressed as mean values obtained in different experiments ± standard error (SE). ** *p* ≥ 0.05. *** *p* ≥ 0.01.

**Figure 3:**
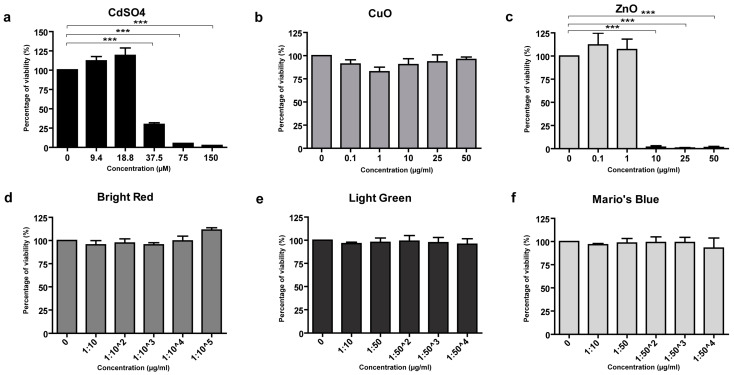
MTS assay of toxicity of NP standards in primary human keratinocytes, and of tattoo inks in HaCaT cells. CdSO_4_ was the positive control (**a**). In primary human keratinocytes, no toxicity was observed with CuO NPs (**b**), whereas a dose-dependent toxicity was seen only with the ZnO NPs (**c**). The three commercial inks tested in HaCaT cells at different dilutions did not show toxicity in this assay (**d**–**f**). Data are expressed as mean values obtained in different experiments ± standard error (SE). *** *p* ≥ 0.01.

To analyze the possible toxicity of metal NP certified reference standards, the MTS assay was performed on HaCaT cells and primary human fibroblasts and keratinocytes. The metal NPs tested were Al_2_O_3_, Cr_2_O_3_, Fe_2_O_3,_ TiO_2_, CuO, and ZnO, while CdSO_4_ was used as a toxicity-positive control (Figure 1a, Figure 2a, Figure 3a). Ag and Au were not analyzed since they were not present in the three tattoo inks. No toxicity was observed in HaCaT cells following treatment with Ag, Al_2_O_3_, Cr_2_O_3_, Fe_2_O_3,_ or with TiO_2_, and ZnO NPs (Figure 1b,d). Only CuO NPs showed dose-dependent toxicity in HaCaT cells (IC50 17 μg/mL) (Figure 1c).

In primary human fibroblasts, toxicity was not observed after treatment with Al_2_O_3_, Fe_2_O_3_, Cr_2_O_3_, or with TiO_2_ NPs (Figure 2b). CuO NPs showed a dose-dependent toxicity (IC50 1.2 μg/mL) (Figure 2c). Furthermore, primary human fibroblasts were sensitive to the highest concentration of ZnO NPs (50 μg/mL) (Figure 2d).

Primary human keratinocytes did not show a toxicity response to treatment with Al_2_O_3_, Cr_2_O_3_, Fe_2_O_3_, and TiO_2_ NPs and, differently from the other cell types analyzed, also showed no response to CuO NPs (Figure 3b). On the contrary, primary keratinocytes were more sensitive to ZnO NPs, showing toxic damage at the concentration of 10 μg/mL (Figure 3c).

Despite the ink colors could interfere with the MTS assay readout, we tested the three chosen inks on HaCaT cells to analyze their possible toxic effects in cultured cells. As shown in Figure 3d–f, none of the three inks had any toxic effect on cultured HaCaT cells at the dilutions used.

### 3.4. Red Ink Tattooing Caused Damages to the Skin

**Figure 4:**
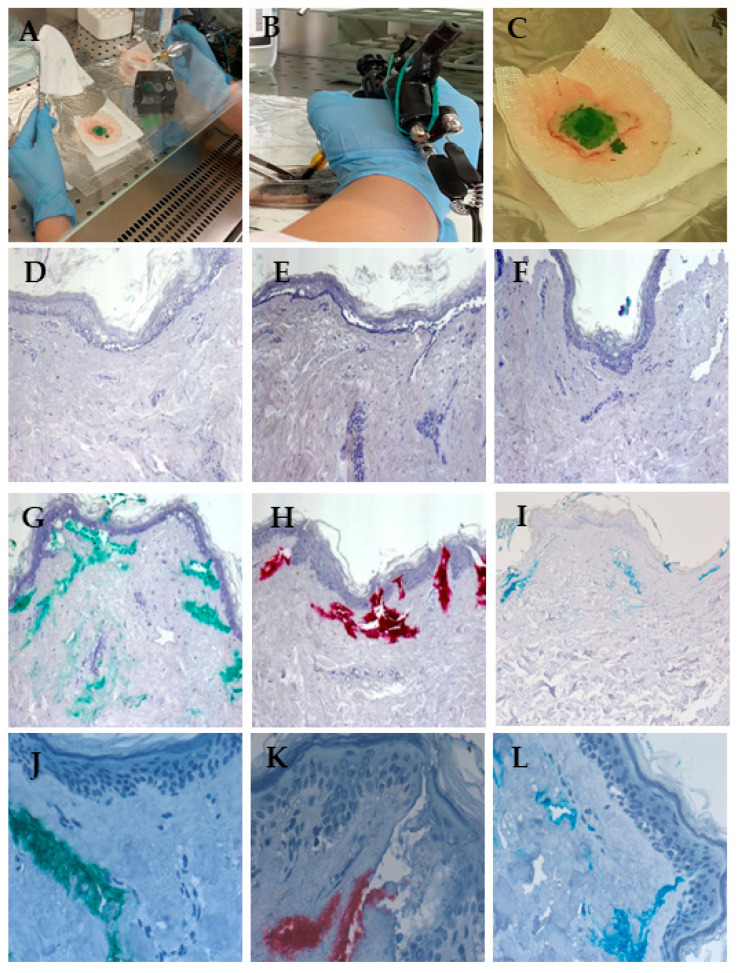
Ex vivo tattooing of human skin explants. (**A**) Photograph of the ex vivo tattooing procedure; (**B**) photograph of the tattoo machine used; (**C**) the final tattooed skin explant. For histology examination of the tattooed skin explants, hematoxylin and eosin staining was performed as described in the Materials and Methods section. (**D**) Skin explant tattooed with a PBS solution; (**E**) skin explant tattooed with a 10 μg/mL solution or (**F**) with a 10 μg/L solution of the CuO NP reference standard; (**G**,**J**) skin explant tattooed with green ink, (**H**,**K**) red ink, or (**I**,**L**) blue ink. Representative images are shown. Magnification: 100× for (**D**–**I**); 400× for (**J**–**L**).

To test the potential toxic effects of tattoo inks and standard metal NPs on human skin, we developed a novel system in which skin explants obtained from plastic surgery patients were tattooed ex vivo using the same machine and inks that may be present in a tattoo studio. As shown in Figure 4A–C, tattooing was performed under sterile conditions using the three inks, Mario’s Blue, Light Green, and Bright Red, used in the MTS assay on HaCaT cells. The metal NPs present in each ink are characterized in Table 3, and the amounts of metal NPs were previously reported [10]. The concentrations of NPs measured were 0.13 and 0.15 μg/g Al_2_O_3_, 0.28 and 0.16 μg/g Cr_2_O_3_, 3287 and 581 μg/g CuO, and 1093 and 1194 μg/g TiO_2_ in the Mario’s Blue and Light Green inks, respectively. On the other hand, the Bright Red ink showed a concentration of 2.26 μg/g Al_2_O_3_ NPs and 0.50 μg/g ZnO NPs. As a negative control, skin explants were injected with 1x PBS to evaluate the mechanical damage. In addition, CuO NPs (25–55 nm), having demonstrated cellular toxicity in both HaCaT and primary human fibroblasts but not in primary human keratinocytes, were tattooed at two different concentrations (10 μg/mL and 10 μg/L) on the skin explants.

After tattooing, the skin explants were left in the culture medium for 72 h. Then, the central part of the tattoo was processed either for SP ICP-MS analysis to identify metal NPs retained in the tissue and assess any physicochemical modifications, or for histology and immunohistochemistry to detect in situ ink localization and possible damage to the skin tissue.

The SP ICP-MS analysis of the tattooed skin explants confirmed the absence of Ag and Au NPs and showed comparable diameters of Cr_2_O_3_ and CuO (<50 nm) NPs in both KBM medium and skin samples, while Fe_2_O_3_ and TiO_2_ NPs showed smaller diameters in skin explants compared to the KBM medium (Table 4), indicating a possible selective presence of smaller NPs in the tissue.

Histological analysis showed that acute cutaneous trauma was observed in all tattooed skin explants, with areas of separation between the epidermis and dermis and accumulation of an inflammatory infiltrate, which was more pronounced when the higher concentration of CuO NPs (10 μg/mL) was used (Figure 4D–I). In addition, explants tattooed with colored ink showed a distribution of pigments deep in the dermis and near blood and lymphatic vessels, indicating a possible pathway for systemic diffusion (Figure 4G–L).

**Figure 5:**
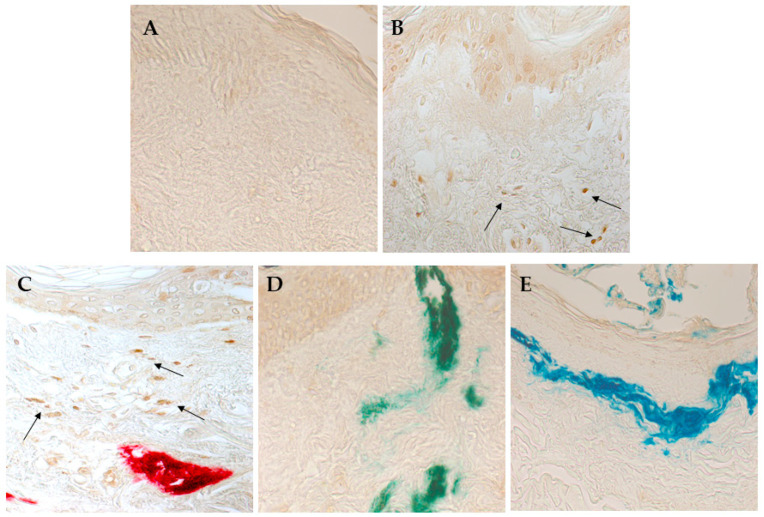
TUNEL assay of tattooed human skin explants. (**A**) Skin explant tattooed with 1× PBS; (**B**) skin explant tattooed with CuO NP reference standard; (**C**) skin explant tattooed with red ink; (**D**) skin explant tattooed with green ink; (**E**) skin explant tattooed with blue ink. Representative images are shown. Arrows indicate the apoptotic cells. Magnification: 400×.

To analyze the possible induction of cell apoptosis by the tattooing procedure or by the standard NPs or colored inks, a TUNEL colorimetric assay was used. As shown in Figure 5, despite the observed mechanical damage, tattooing with the PBS solution did not induce apoptosis in skin cells (Figure 5A). On the other hand, tattooing with the standard CuO NPs and the red ink induced apoptosis in the dermis (Figure 5B,C, respectively). No significant signals of ink-induced apoptosis were observed for the green and blue inks (Figure 5D,E, respectively).

**Figure 6:**
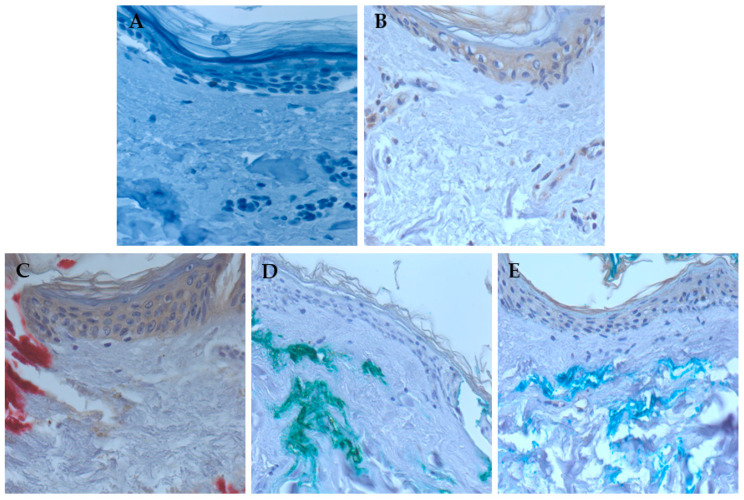
Immunohistochemical analysis of tattooed human skin explants with an anti-IL-8 antibody. Anti-IL-8 antibody staining of skin explants tattooed with PBS (**A**), CuO standard nanoparticles (**B**), red ink (**C**), green ink (**D**), and blue ink (**E**). Representative images are shown. Magnification: 400×.

Immunohistochemical analysis was also performed on the tattooed skin explants to detect the expression of inflammatory chemokines. As shown in Figure 6, tattooing with CuO NPs, with the red ink, and, to some extent, with the blue ink induced the expression of IL-8 in the epidermis. 

## 4. Discussion

Assessing the skin toxicity of metal NPs in tattoo inks is still a challenge. This study investigated the possible skin toxicity of metal NPs associated with tattooing using both certified reference standards of metal NPs and real samples of tattoo inks, along with in vitro tests and ex vivo tattooing of skin explants.

Particle size is a critical characteristic of NPs that can significantly affect important particle properties, such as reactivity, absorption, and toxicity [24]. In healthy individuals, NPs with diameters smaller than 45 nm are able to penetrate the skin barrier, while larger particles are unable to cross the barrier [25]. In this study, the size characterization of reference standards of Ag, Al_2_O_3_, Au, Cr_2_O_3_, CuO, Fe_2_O_3_, TiO_2_, and ZnO NPs was performed using DLS and SP-ICP-MS. DLS is a fast and simple tool used to measure particle size, size distribution, and stability in suspensions [26]. In contrast, SP ICP-MS is a more efficient technique that allows the identification of elemental composition and concentration, as well as the size measurement of metal NPs [10,20]. The difference in the measured NP size between DLS and SP ICP-MS can be attributed to the fact that DLS measures the hydrodynamic diameter, which also includes the surrounding layer of adsorbed molecules (water, salts, or proteins from the dispersant), while SP ICP-MS provides a measurement of the core size of the metal NPs [27]. The differences in aggregation tendencies, surface reactivity, and hydration effects contribute to the broader size distributions, higher PDI values, and less reproducible results in DLS measurements of metal NPs [28]. Dispersion in DMEM can lead to the adsorption of proteins on the NP surface and the formation of a biomolecule corona, which drives much of the interactions with cells [29]. It is, therefore, crucial to ensure that NPs’ stability is also maintained when dispersed in such a medium before testing their effects on cells and after the incubation time. On the other hand, time-dependent Z-average changes were observed, with smaller values at 24 h. These results suggested that metal NPs undergo some dissolution in the cell culture medium over time [30]. On the other hand, SP ICP-MS showed good agreement with the certified diameters for Ag, Al_2_O_3_, Cr_2_O_3_, CuO, Fe_2_O_3_, TiO_2_, and ZnO NPs. A lower size accuracy was determined for Au due to the proximity of the certified size to its respective limit of detection [20]. SP ICP-MS, differently from DLS, is not as strongly sensitive to the presence of larger particles or aggregates. Changing the dispersant (water or cell medium) did not visibly influence the measurement of NP size. The analysis of the tattoo inks showed the presence of Al_2_O_3_, Cr_2_O_3_, CuO, Fe_2_O_3_, TiO_2_, and ZnO NPs, with diameters ranging between 45.2 nm and 260.2 nm and concentrations between 0.13 μg/g and 3287 μg/g, as previously observed [10].

The selected concentrations for the cellular viability assay of the NP reference standards represent a dose range reflecting realistic exposure scenarios, considering the intrinsic limits of in vitro treatments (such as NP dispersion in cell culture media or in vitro dosimetry).

The MTS protocol used in this study was adapted from NP viability testing in the EU project NanoValid (http://www.nanovalid.eu/index.php/sops-standard-operating-procedures, accessed on 16 December 2019). Therefore, the selected concentrations represent a range of realistic exposure scenarios, considering the intrinsic limits of in vitro tests such as NP dispersion in liquid culture media and the ratio of the number of particles to the surface area of the culture vessel. Metal ions, such as iron, manganese, copper, and zinc, play fundamental roles in various cellular processes by serving as essential cofactors of enzyme systems. Their availability regulates cellular metabolism and can also affect the availability of other trace metals and the stability of some medium components. That may be why slight increases in cell viability compared to the control were observed in the MTS assay with a hormesis-like effect [31]. It is also well known that colorimetric cytotoxicity assays, such as the MTS assay, have some drawbacks, including possible interference from the tested NPs [31,32]. The most frequently reported limitations are NPs’ interference with the assay absorption wavelength or their interaction with assay reagents, such as the reduction of the tetrazolium compound, which generates colored formazan products in the absence of cellular activity. Moreover, some metal NPs, such as gold or platinum, can catalyze redox reactions [33]. To avoid false positive results, our assay scheme included some cell-free wells in which NPs were in contact with the assay components alone.

When analyzing the possible skin toxicity induced by the reference standard metal NPs in 2D cell cultures, the three cell types tested—HaCaT cells, primary fibroblasts, and primary keratinocytes—showed different susceptibilities to NP treatment. Indeed, HaCaT cells, a spontaneously transformed aneuploid immortal keratinocyte cell line from adult human skin [34], exhibited toxicity upon treatment with CuO NPs. Similar toxicity due to CuO NPs was observed in primary fibroblasts but not in primary keratinocytes. Although derived from human keratinocytes, HaCaT cells have a homozygous mutation in the gene encoding p53, making them more susceptible to DNA damage. In addition, HaCaT cells differ from normal keratinocytes in membrane lipid composition and differentiation ability [35]. Thus, CuO NPs may affect these differences, inducing pronounced toxicity in HaCaT cells but not in normal keratinocytes. Conversely, in contrast to what is reported in the literature [36,37], HaCaT cells showed no toxicity when treated with ZnO NPs, whereas both primary fibroblasts and keratinocytes showed a toxic response to ZnO NPs—fibroblasts at the highest concentration used and keratinocytes at a dose-dependent concentration starting from 10 μg/ml. Explaining these differences requires further evaluation. The three commercial inks did not show any toxicity in HaCaT cells. Other authors have investigated tattoo ink toxicity in HaCaT cells using a cell viability assay and analysis of IL-18 release [38]. They reported a dose- and color-dependent reduction in viability, together with an increase in IL-18 secretion in the culture medium. It should be noted that tattoo inks are industrial products with contaminants and compositional variations between brands and batches, and these variables may account for the contrasting data we obtained. Furthermore, our results suggest that HaCaT cells may not be the most appropriate cell type for in vitro testing of tattoo inks.

Using the novel ex vivo model of human tattooed skin explants, we observed that the red tattoo ink was the most dangerous. Clinical case reports have already pointed out that red tattoo ink is the most commonly involved in the development of tattoo complications [39,40]. In some cases, the development of squamous cell carcinoma was reported as a consequence of red tattoos [41]. An increase in apoptotic cells in the dermis and induction of IL-8 expression in the epidermis were also found with the application of red tattoo ink. The analysis of the metal NPs’ composition in red tattoo ink revealed that this ink presented a higher ZnO NP concentration compared to the green and blue tattoo inks.

Nano-ZnO is one of the most commonly used nanomaterials, with main applications such as polymer fillers and UV absorbers. The potential toxicity of Zn NPs in the red ink raises concerns for the use of ZnO NPs in sunscreens when applied to damaged skin, where penetration in the lower dermis is not hindered by a whole stratum corneum. Zn traces were found in the blood and urine of volunteers after 5 days of application of sunscreens containing ZnO NPs [42]. In addition, ZnO NPs can release a great number of ions [25]. For these reasons, the Zn NPs found in the red tattoo ink could be responsible for the skin damage we observed and may be a source of Zn ions for systemic exposure.

The ex vivo tattooed skin model here described shows improvements with respect to the previously proposed methods. Model organisms such as *Daphnia magna* and *Xenopus laevis* have been used for assessing the toxicity of commercially available tattoo inks [43,44]. In these models, red tattoo ink was also the most toxic, although the authors underlined that the higher toxicity could have been due to the azo compounds in the pigment rather than the metal NP composition [43]. A reconstructed human full-thickness skin model named TatS was also established, where tattoo ink pigments were incorporated into the similar dermis [45]. In our ex vivo model, we maintained the skin tissue integrity and injected the tattoo inks with the same needles used by professional tattoo artists, also providing the mechanical damage due to needle insertion. However, the mechanical damage due to the multiple small holes created by the millimeter-sized solid needles in the present model may not accurately represent real skin damage because the skin explants lacked subcutaneous tissue, which could better absorb such mechanical insult. On the contrary, accumulation of an inflammatory infiltrate was observed in this model, but this could be underestimated due to the lack of blood supply to the excised tissue explant. The other two phases of healing, tissue formation and tissue remodeling, could not be observed because the tattooed skin explants were maintained in culture for only 72 h. Nevertheless, our ex vivo model could better represent the in vivo situation with respect to other model systems. Lin et al. used a model of reconstructed human full-thickness skin that was tattooed in vitro similarly our ex vivo model [46]. In their in vitro model, macrophages were added to fibroblasts in a collagen matrix to reconstruct a similar dermis. The study mainly focused on the mechanisms of ink uptake by the macrophages, and no data on skin toxicity were reported, making it difficult to compare the two models.

In conclusion, the present results indicate that tattooing can involve exposure to toxic metal NPs and skin damage. The metal NP composition should be carefully evaluated to avoid exposure to toxic metal NPs present in specific colored tattoo inks. Moreover, the use of ex vivo tattooed human skin explants, maintaining the original tissue architecture, proved to be more advantageous for studying the skin toxicity of tattoo inks.

## Figures and Tables

**Table 1:** Instrument settings for DLS and SP ICP-MS.

DLS	
Instrument	Zetasizer (Malvern Panalytical, Malvern, UK)
Record number	10
Temperature	25 °C
Dispersant refractive index/viscosity (cP)	Water, 1.330/0.8872 cP; DMEM, 1.330/0.9400 cP
Material refractive index/absorption	Ag, 0.18/0.010; Al_2_O_3_, 1.75/0.430; Au, 0.20/0.500; Cr_2_O_3_, 2.50/0.800; CuO, 0.86/0.737; Fe_2_O_3_, 2.94/5.200; TiO_2_, 2.49/0.100; ZnO, 2.00/0.002; polystyrene, 1.59/0.01
**SP ICP-MS**	
Instrument	iCAP-Q (Thermo Fisher, Waltham, MA USA)
Nebulizer	Quartz concentric
Spray chamber	Quartz cyclonic
Sample uptake rate	0.33 mL/min
RF power	1450 W
Masses	^107^Ag, ^197^Au, ^27^Al, ^52^Cr, ^63^Cu, ^56^Fe, ^48^Ti, ^66^Zn
Acquisition mode and time	Q-Cell in KED (4.8 mL/min He), 60 s
Nebulization efficiency	4%
Dwell time	5 ms

**Table 2:** Size characterization of metal NP certified reference standards by DLS and SP ICP-MS.

NPs	Certified Diameter	Dispersant	Time	DLS		SP ICP-MS
	(nm)		(h)	Z-Average (nm)	PDI	Diameter (nm)
Ag	20 nm	H_2_O	0	24.9	0.104	27.3 ± 1.1
		DMEM	0	20.2	0.377	23.7 ± 2.0
		DMEM	24	15.7	0.238	20.3 ± 1.3
Al_2_O_3_	30 nm	H_2_O	0	166.1	0.140	38.4 ± 8.2
		DMEM	0	196.3	0.600	45.4 ± 9.6
		DMEM	24	185.1	0.726	34.3 ± 9.7
Au	5 nm	H_2_O	0	27.3	0.375	14.4 ± 2.3
		DMEM	0	23.2	0.526	14.8 ± 2.1
		DMEM	24	17.2	0.450	13.9 ± 2.3
Cr_2_O_3_	60 nm	H_2_O	0	181.1	0.466	60.1 ± 35.0
		DMEM	0	134.5	0.717	61.1 ± 37.5
		DMEM	24	86.4	0.727	59.3 ± 17.9
CuO	25–55 nm	H_2_O	0	227.1	0.203	43.3 ± 11.7
		DMEM	0	243.0	0.338	41.3 ± 7.9
		DMEM	24	192.4	0.467	40.3 ± 5.5
Fe_2_O_3_	30 nm	H_2_O	0	165.9	0.142	43.6 ± 12.5
		DMEM	0	300.5	0.358	49.4 ± 10.2
		DMEM	24	287.5	0.314	47.7 ± 11.3
TiO_2_	<100 nm	H_2_O	0	310.3	0.489	89.6 ± 12.2
		DMEM	0	100.7	0.743	103.9 ± 20.2
		DMEM	24	80.6	0.975	99.9 ± 19.1
ZnO	30–40 nm	H_2_O	0	432.1	1.000	31.6 ± 4.5
		DMEM	0	43.7	0.740	37.1 ± 3.8
		DMEM	24	31.9	0.461	35.6 ± 4.6

**Table 3:** Tattoo ink characterizations by DLS and SP ICP-MS.

	Bright Red		Light Green		Mario’s Blue	
**DLS**	**Z-Average (nm)**	**PDI**	**Z-Average (nm)**	**PDI**	**Z-Average (nm)**	**PDI**
	206.7	0.347	193.4	0.176	192.8	0.238
**SP ICP-MS**	**Diameter (nm)**		**Diameter (nm)**		**Diameter (nm)**	
Ag	nd		nd		nd	
Al_2_O_3_	137.8 ± 20.1		115.2 ± 13.8		115.7 ± 13.1	
Au	nd		nd		nd	
Cr_2_O_3_	55.0 ± 4.5		62.3 ± 4.0		45.2 ± 7.3	
CuO	nd		45.8 ± 13.4		57.5 ± 9.2	
Fe_2_O_3_	75.5 ± 8.5		77.2 ± 11.0		77.5 ± 11.6	
TiO_2_	166.1 ± 34.2		260.2 ± 30.5		228.4 ± 53.6	
ZnO	26.2 ± 5.9		nd		nd	

nd: not detected.

**Table 4:** Metal NP characterization in tattooed skin explants.

		Bright Red	Light Green	Mario’s Blue
**Nanoparticles**	**Dispersant**	**Measured Diameter**	**Measured Diameter**	**Measured Diameter**
		**(nm)**	**(nm)**	**(nm)**
Ag	Skin	nd	nd	nd
	KBM	nd	nd	nd
Al_2_O_3_	Skin	50.2 ± 8.9	49.8 ± 5.0	46.1 ± 4.1
	KBM	175.7 ± 18.3	190.9 ± 12.8	136.0 ± 13.4
Au	Skin	nd	nd	nd
	KBM	nd	nd	nd
Cr_2_O_3_	Skin	50.2 ± 7.4	52.2 ± 6.5	41.1 ± 7.0
	KBM	52.1 ± 8.0	50.0 ± 6.7	40.2 ± 7.2
CuO	Skin	nd	43.7 ± 8.3	53.2 ± 9.9
	KBM	nd	46.6 ± 6.1	44.3 ± 7.2
Fe_2_O_3_	Skin	39.4 ± 5.2	33.6 ± 3.9	36.9 ± 6.0
	KBM	74.9 ± 8.9	79.0 ± 9.5	84.9 ± 11.6
TiO_2_	Skin	118.4 ± 23.7	162.8 ± 35.4	177.2 ± 17.6
	KBM	165.7 ± 44.7	233.9 ± 37.7	210.4 ± 34.3
ZnO	Skin	22.7 ± 5.9	nd	nd
	KBM	26.2 ± 6.2	nd	nd

nd: not detected.

## Data Availability

The original contributions presented in the study are included in the article. Further inquiries can be directed to the corresponding author.
